# Investigating Urinary Pathogens and Their Antibiotic Resistance: A Cross-Sectional Urine Culture Study

**DOI:** 10.7759/cureus.63663

**Published:** 2024-07-02

**Authors:** Muzamil Khan, Fazeel Hussain, Muhammad Naseem, Ruchira Clementina, Nida Gul, Aysha Habib, Laiba Ali Khan, Ayaz Ali, Waqas Rahim, Izhar Khan

**Affiliations:** 1 Internal Medicine, The George Washington University School of Medicine and Health Sciences, Washington, D.C., USA; 2 Internal Medicine, Bangladesh Medical College, Dhaka, BGD; 3 Neuro-Rehab, Musgrove Park Hospital, Taunton, GBR; 4 Medicine, Government Medical College, Nizamabad, IND; 5 Internal Medicine, MTI Khyber Teaching Hospital, Peshawar, PAK; 6 Internal Medicine, Saidu Medical College, Swat, PAK; 7 General Surgery, MTI Khyber Teaching Hospital, Peshawar, PAK

**Keywords:** proteus mirabilis, uropathogens, escherichia coli, antimicrobial resistance, urinary tract infection

## Abstract

Introduction: Urinary tract infections (UTIs) are among the most prevalent infectious diseases. Females are more affected than males. The primary culprit is Escherichia coli. Multiple research investigations have documented widespread antimicrobial resistance in uropathogens, sparking global concerns, especially regarding the rise of multidrug resistance (MDR).

Methodology: This cross-sectional study was conducted from December 2023 to March 2024. A non-probability purposive sampling technique was employed to select participants, and informed consent was obtained from them. Data were extracted from the culture and sensitivity reports of these patients. The collected data were meticulously entered into IBM SPSS Statistics for Windows, Version 21 (IBM Corp., Armonk, NY). The findings were then presented using a blend of percentages and numerical figures, offering a clear and concise representation of the data.

Results: Our study of 313 participants showed a higher prevalence of UTIs in females (219, 70%) compared to males (94, 30%). E. coli and Citrobacter were the predominant pathogens, with E. coli and Citrobacter more common in females, while Enterobacter and Staphylococcus were more prevalent in males. Antibiogram analysis revealed sensitivities to specific drugs like nitrofurantoin and meropenem, while resistance was observed against others, including polymyxin B and ampicillin. These findings stress the need for tailored UTI treatment approaches.

Conclusions: In conclusion, our research highlights a concerning trend of escalating antibiotic resistance among Pakistani patients with UTIs. Tobramycin B, ticarcillin-clavulanic acid, ampicillin, and clotrimazole exhibited the highest resistance rates, while imipenem, meropenem, nitrofurantoin, sulfonamides, and tigecycline demonstrated notable sensitivity. These findings emphasize the urgent need for the exploration of alternative treatment options to combat rising resistance levels effectively.

## Introduction

One of the most common infectious disorders is urinary tract infection (UTI). With an estimated yearly global incidence of at least 250 million cases, UTIs are a common illness that doctors encounter in developing nations [1,2]. At any age, UTIs tend to affect both genders equally. However, due to their anatomical makeup or the high bacterial load in the urothelial mucosa, women are more likely than men to get UTIs. In addition to these factors, pregnancy, urinary tract blockage, and sexual activity all play a role in the development of UTIs in females. One in two women experiences a UTI at some point in their lives. According to reports, 30% of children worldwide suffer from UTIs. In Iran, it has been documented that approximately 1% of boys and 3% of girls experience their initial occurrence of UTI before reaching the age of 11 years [3].

Several types of gram-positive and gram-negative bacteria contribute to UTIs, but the primary culprit is Escherichia coli, a gram-negative, facultative anaerobic bacterium known for its uropathogenic properties [4]. Besides E. coli, UTIs commonly involve other microbes like Klebsiella spp., Acinetobacter baumannii, and Pseudomonas aeruginosa. Moreover, gram-positive bacteria, such as Methicillin-resistant Staphylococcus aureus (MRSA) and Staphylococcus saprophyticus, are also implicated in UTI occurrences [5].

The approach to treating UTI cases typically involves initiating empirical therapy, guided by knowledge derived from the antimicrobial resistance profile of the urinary pathogens [6]. Multiple research investigations have documented widespread antimicrobial resistance in uropathogens, sparking global concerns, especially regarding the rise of multidrug resistance (MDR) and extended-spectrum beta-lactamases (ESBLs) [7,8].

The growing challenge of antibiotic resistance among urinary tract bacteria presents a dynamic regional shift. Thus, it becomes imperative to discern which antibiotics have encountered resistance. This knowledge equips healthcare professionals with vital guidance for prescribing medications, ensuring informed decisions, and providing effective patient care. This study is motivated by the pressing need to navigate this evolving antibiotic resistance terrain, aiming to elevate healthcare standards and optimize treatment strategies.

## Materials and methods

This cross-sectional study was conducted within the departments of General Medicine, Nephrology, and Urology at Khyber Teaching Hospital (KTH) in Peshawar, spanning from December 2023 to March 2024. Formal permission for data collection was obtained from the Medical Director of KTH and the Director of Medical Education at Khyber Medical College (Ref: 280/DME/KMC), ensuring adherence to ethical guidelines. All patients admitted to these departments with UTIs during the specified period were eligible for inclusion. A non-probability purposive sampling technique was employed to select participants, and informed consent was obtained from them. Patients with catheterization were excluded from the study to maintain the integrity and validity of the findings, as their urine samples could potentially be contaminated with bacteria from the catheter itself.

Procedure for bacterial isolation and identification

An early morning midstream urine sample was taken from UTI patients who have not used antibiotics for the past 48-72 hours and do not have catheters passed. Total samples were taken from 400 patients. The isolation of uropathogens was conducted using a surface streak method on both blood and MacConkey agar (Oxoid Ltd., Basingstoke, Hampshire, UK) with calibrated loops for a semiquantitative approach. These were incubated aerobically at 37 °C for 24 hours. Cultures that showed no growth after 24 hours were incubated for an additional 48 hours. A specimen was deemed positive for a UTI if a single organism was cultured at a concentration of ≥10^5^ CFU/mL. Bacterial identification was carried out using a series of biochemical tests, including indole, citrate utilization, oxidase activity, hydrogen sulfide production, lysine decarboxylase activity, lactose fermentation, urease activity, gas production, catalase activity, coagulase activity, mannitol fermentation, and susceptibility to novobiocin [9].

Susceptibility testing

Antibiotic susceptibility was carried out on Müller-Hinton agar and blood agar. The antimicrobial susceptibility of the isolated bacterial uropathogens was assessed using the disk diffusion method, following the guidelines provided by the Clinical Laboratory Standards Institute (CLSI) [10]. Antibiotic discs used were nitrofurantoin, fosfomycin, piperacillin + tazobactam, meropenem, imipenem, cefoperazone/sulbactam, nalidixic acid, ampicillin, trimethoprim, ceftriaxone, amikacin, aztreonam, ciprofloxacin, co-amoxiclav, clotrimazole, cefuroxime, chloramphenicol, ceftazidime, gentamicin, levofloxacin, gatifloxacin, cefixime, ticarcillin-clavulanic acid, ertapenem, cefpirome, colistin, polymyxin B, tobramycin, minocycline, cefepime, ofloxacin, norfloxacin, tigecycline, Cefobid (cefoperazone), cefotaxime, sulfonamides, doxycycline, and maxipime. A total of 313 patients showed growth of UTI-causing bacteria.

Data analysis

The collected data were meticulously entered into IBM SPSS Statistics for Windows, Version 21 (IBM Corp., Armonk, NY), a powerful statistical analysis tool, to explore the resistance and sensitivity profiles of the organisms under study. The chi-square test was applied to find out the relationship between UTI and gender. A *P*-value less than 0.05 was considered significant, while a *P*-value greater than 0.05 was considered insignificant. The findings were then presented using a blend of percentages and numerical figures, offering a clear and concise representation of the data. This approach not only enhances the accessibility of the results but also facilitates a deeper understanding of the intricate patterns observed in antimicrobial resistance.

## Results

**Figure 1:**
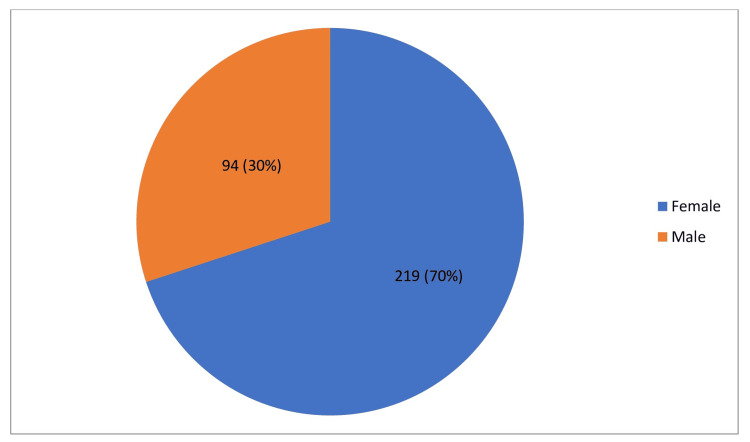
Gender-wise prevalence.

In our study, 400 patients participated, and 313 of them showed bacterial growth indicative of a UTI. Out of these 313 patients, 94 (30%) were male, while 219 (70%) were female. To determine the association between UTI occurrence and gender, we applied the chi-square test. The results showed a significant difference, with a *P*-value of less than 0.001. This statistical outcome indicates that the prevalence of UTIs is significantly higher among females compared to males (Figure 1).

**Figure 2:**
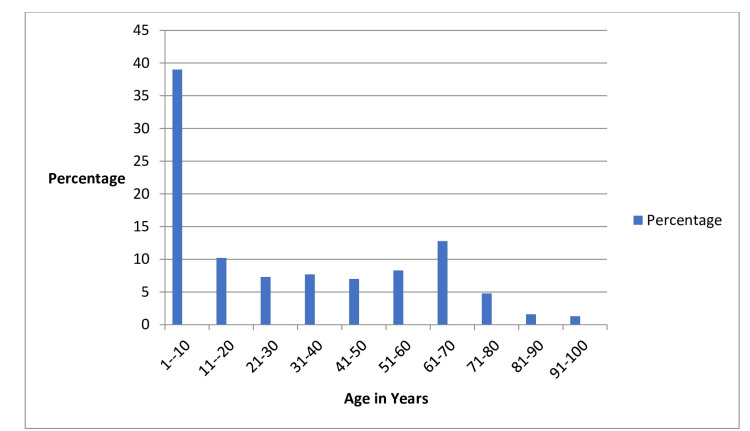
Age-wise prevalence.

In our study, the participants' ages ranged from 1 to 97 years. Among the 313 patients who exhibited bacterial growth indicative of a UTI, a notable observation was that the majority fell within the age group of 1-10 years, suggesting that younger patients, particularly those in early childhood, were more commonly affected by UTIs in our study. Further analysis revealed that within this age range, both males and females showed bacterial growth, although the overall prevalence remained higher among females. This age-specific trend underscores the importance of monitoring and addressing UTIs in pediatric population. Age-specific prevalence is illustrated in Figure 2 and Table 1.

**Table 1:** Gender-wise prevalence. The chi-square test was applied. *P < 0.05 was considered significant, and *P* > 0.05 was considered nonsignificant.

Male, *n* (%)	Female, *n* (%)	*P*-value*
94 (30)	219 (70)	<0.001

Among the 313 patients who showed bacterial growth indicative of a UTI, approximately 154 (49.2%) were found to have E. coli, making it the most prevalent bacterium. This was followed by Citrobacter in 82 patients (26.2%), Morganella morganii in 39 patients (12.5%), Serratia species in 11 patients (3.5%), Pseudomonas in 10 patients (3.2%), Enterobacter in 8 patients (2.6%), Providencia in 4 patients (1.3%), Staphylococcus in 3 patients (1%), and Proteus in 2 patients (0.6%).

Further analysis revealed that the occurrence of E. coli and Citrobacter was notably higher in female patients, aligning with the overall trend of higher UTI prevalence among females in the study. Conversely, bacteria such as Enterobacter and Staphylococcus were found more frequently in male patients. This gender-specific distribution of bacterial growth highlights the need for targeted approaches in the diagnosis and treatment of UTIs, taking into consideration the differing bacterial profiles between male and female patients. Details are given in Table 2.

**Table 2:** Age-wise prevalence.

Age of participant (Years)	Male, *n* (%)	Female, *n* (%)	Total, *n* (%)
1-10	28 (8.9)	94 (30)	122 (30.5)
11-20	12 (3.8)	20 (6.3)	32 (8.0)
21-30	4 (1.3)	19 (6.1)	23 (5.8)
31-40	6 (1.9)	18 (5.7)	24 (6.0)
41-50	5 (1.6)	17 (5.4)	22 (5.5)
51-60	9 (2.8)	17 (5.4)	26 (6.5)
61-70	15 (4.8)	25 (7.9)	40 (10.0)
71-80	10 (3.1)	5 (1.6)	15 (3.8)
81-90	5 (1.6)	0 (0)	5 (1.3)
91-100	0 (0)	4 (1.2)	4 (1.0)

The results of the antibiogram analysis provided important insights into the drug sensitivities and resistances observed among the UTI-causing bacteria. Several antibiotics demonstrated notable effectiveness against these pathogens. Specifically, nitrofurantoin, fosfomycin, piperacillin/tazobactam, meropenem, imipenem, cefoperazone/sulbactam, amikacin, ertapenem, amoxicillin, tigecycline, and sulfonamides were identified as having high sensitivities. This indicates that these drugs were generally effective in inhibiting the growth of the UTI bacteria identified in our patient cohort.

On the other hand, certain antibiotics exhibited higher resistance rates among the bacteria. Notably, norfloxacin, Cefobid (cefoperazone), minocycline, polymyxin B, ticarcillin-clavulanic acid, and ampicillin showed lower efficacy, with the bacteria demonstrating substantial resistance to these medications. This resistance pattern suggests that these antibiotics may be less suitable for treating UTIs in the studied population.

**Figure 3:**
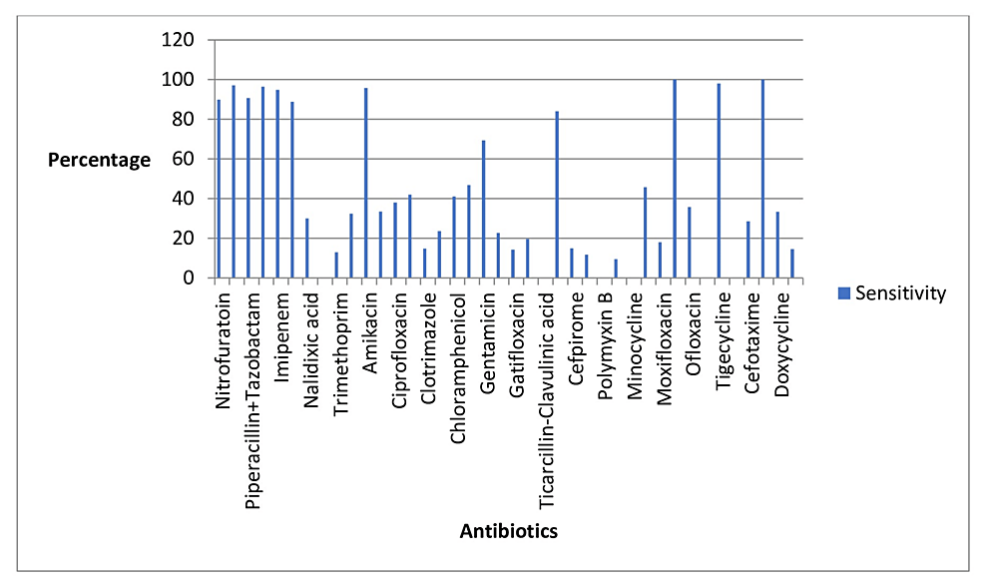
UTI antibiogram sensitivity profile. UTI, urinary tract infection

**Figure 4:**
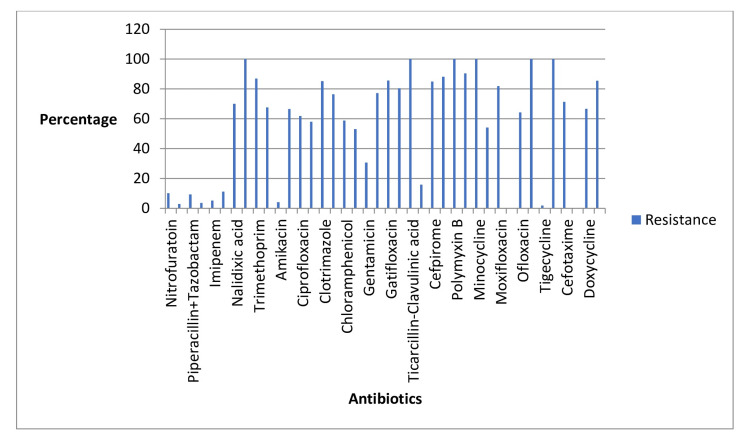
Antibiotics and their resistance profiles.

The findings underscore the importance of conducting antibiogram analyses to inform the selection of appropriate antimicrobial therapy. By identifying which antibiotics are more likely to be effective and which are prone to resistance, healthcare providers can make more informed decisions, leading to better patient outcomes and more effective management of UTIs. Details are given in Tables 3-4 and Figures 3-4.

**Table 3:** Prevalence of bacteria in patients with UTI. UTI, urinary tract infection

Bacteria name	Frequency (*n*)	Percentage (%)	Male, *n* (%)	Female, *n* (%)	Total male patients, *n* (%)	Total female patients, *n* (%)
E. coli	154	49.2	41 (13.1)	113 (36.1)	41 (43.6)	113 (51.6)
Citrobacter	82	26.2	18 (5.7)	64 (20.4)	18 (19.1)	64 (29.2)
Enterobacter	8	2.6	8 (2.5)	0 (0)	8 (8.5)	0 (0)
Pseudomonas	10	3.2	2 (0.6)	8 (2.5)	2 (2.1)	8 (3.7)
Morganella morganii	39	12.5	19 (6.1)	20 (6.3)	19 (20.2)	20 (9.1)
Staphylococcus	3	1.0	3 (0.9)	0 (0)	3 (3.2)	0 (0)
Proteus	2	0.6	1 (0.3)	1 (0.3)	1 (1.1)	1 (0.5)
Serratia species	11	3.5	2 (0.6)	9 (2.8)	2 (2.1)	9 (4.1)
Providencia species	4	1.3	0 (0)	4 (1.2)	0 (0)	4 (1.8)

**Table 4:** Antibiotics and their sensitivity and resistance.

Antibiotic	Number tested	Sensitivity (S, %)	Resistance (R, %)
Nitrofurantoin	237	89.87	10.12
Fosfomycin	208	97.11	2.88
Piperacillin + Tazobactam	312	90.70	9.29
Meropenem	310	96.45	3.54
Imipenem	310	94.83	5.16
Cefoperazone/Sulbactam	295	88.81	11.18
Nalidixicacid	40	30	70
Ampicillin	143	0	100
Trimethoprim	23	13.01	86.95
Ceftriaxone	179	32.40	67.59
Amikacin	289	95.84	4.15
Aztreonam	296	33.44	66.55
Ciprofloxacin	305	38.03	61.96
Co-amoxiclav	276	42.02	57.97
Clotrimazole	196	14.79	85.20
Cefuroxime	72	23.61	76.38
Chloramphenicol	180	41.11	58.88
Ceftazidime	301	46.84	53.15
Gentamicin	297	69.36	30.63
Levofloxacin	119	22.68	77.31
Gatifloxacin	21	14.28	85.71
Cefixime	92	19.56	80.43
Ticarcillin-Clavulanic acid	15	0	100
Ertapenem	113	84.07	15.92
Cefpirome	80	15	85
Colistin	17	11.76	88.23
PolymyxinB	15	0	100
Tobramycin	63	9.52	90.47
Minocycline	15	0	100
Cefepime	286	45.80	54.19
Ofloxacin	42	35.71	64.28
Norfloxacin	9	0	100
Tigecycline	261	98.08	1.91
Cefobid	30	0	100
Cefotaxime	203	28.57	71.42
Sulfonamides	3	100	0
Doxycycline	6	33.33	66.66
Maxipime	62	14.51	85.48

## Discussion

The rise of antibiotic resistance is becoming a significant global issue. UTIs, which impact many people each year, are caused by bacteria exhibiting a range of resistance to different antibiotics [11]. Hence, identifying the resistance and sensitivity profiles of antibiotics in patients with UTIs is crucial. This knowledge enables healthcare providers to select the most effective treatment, ensuring better patient outcomes and helping to combat the growing issue of antibiotic resistance.

The prevalence of UTIs was higher in females than in males in our study, consistent with the findings of other research [12]. The higher prevalence of UTIs among females can be attributed to several factors that predispose women to these infections [13].

Our study reveals that E. coli is the most common organism responsible for UTIs, a finding that aligns with research conducted in both Iran [14] and Scotland [15]. While various studies indicate that E. coli is typically followed by Klebsiella in prevalence, our research identified Citrobacter as the second most common organism causing UTIs [16]. These variations depend on multiple factors, including geographic region, ethnicity, types of pollution, and other environmental influences [17]. Understanding these variations is crucial for developing targeted treatment strategies and preventive measures. The identification of Citrobacter as a significant UTI pathogen in our study suggests that regional microbial profiles should be closely monitored to ensure effective clinical management. Furthermore, the impact of environmental factors on pathogen prevalence underscores the need for comprehensive public health approaches that address not only medical treatment but also environmental and societal conditions. Future research should aim to explore these factors in greater depth to enhance our understanding of UTI etiology and improve patient outcomes across different regions.

In our study, E. coli and Citrobacter are more prevalent in females than in males, while Staphylococcus and Enterobacter are higher in male patients. A study from China also shows that E. coli is common in females [18].

In our study, the highest resistance was developed to ampicillin, which is consistent with the findings of other research [19, 20]. This widespread resistance can be attributed to the extensive and prolonged use of ampicillin in clinical settings, leading to selective pressure and the proliferation of resistant bacterial strains.

Our study shows higher sensitivity to amoxicillin, while other studies show resistance to it [19]. This discrepancy may be due to regional differences in antibiotic prescribing practices or variations in local bacterial populations. Additionally, variations in resistance patterns can arise from differences in study design, sample size, and the specific patient populations being studied. Understanding these variations highlights the importance of local antimicrobial susceptibility testing to guide effective antibiotic therapy.

Our study identified a notable resistance to polymyxin B, contrasting with other research that reports sensitivity to this antibiotic in multi-drug-resistant pathogens [21-23]. This unexpected finding raises serious concerns about evolving resistance patterns and suggests that local variations in antibiotic usage and resistance mechanisms may be at play. The emergence of resistance to polymyxin B, a critical last-resort antibiotic, underscores the urgent need for enhanced surveillance and innovative approaches to combat antimicrobial resistance. Our findings highlight the importance of continuous monitoring and adapting treatment strategies to effectively address the dynamic landscape of bacterial resistance.

Limitations

The primary limitation of our study is that it was conducted at a single center, Khyber Teaching Hospital. Additionally, the study duration was relatively short, spanning only four months.

## Conclusions

Our study of 313 patients highlights a significant prevalence of UTIs among females compared to males. E. coli and Citrobacter were predominant in females, while Enterobacter and Staphylococcus were more prevalent in males. Antibiotic sensitivity analysis revealed promising results for nitrofurantoin, fosfomycin, and others, while resistance was notable against norfloxacin, polymyxin B, and others. These findings emphasize the need for targeted antibiotic therapy based on local resistance patterns to effectively manage UTIs.
